# Comparison of the Modified Aldrete Score and White Fast-Track Score in the Determination of Post-Anaesthesia Care Unit Discharge Readiness After General Anaesthesia

**DOI:** 10.7759/cureus.112608

**Published:** 2026-07-13

**Authors:** Aatish Mahajan, Shikha Sharma, Varsha Gupta, Abhinav Gupta

**Affiliations:** 1 Department of Anaesthesiology, Critical Care and Pain Medicine, Acharya Shri Chander College of Medical Sciences and Hospital, Jammu, IND; 2 Department of Anaesthesiology, Maharishi Markandeshwar (Deemed to be University), Mullana, IND; 3 Department of Gastroenterology, Maharishi Markandeshwar (Deemed to be University), Mullana, IND

**Keywords:** discharge criteria, general anesthesia, modified aldrete score, post-anesthesia care unit, recovery assessment, white fast-track score

## Abstract

Background: Safe and timely discharge from the post-anaesthesia care unit (PACU) is essential for optimal recovery and efficient resource utilization. This study compared the Modified Aldrete Score (MAS) and White Fast-Track Score (WFTS) in the assessment of PACU discharge readiness, using the conventional 60-minute time-based discharge criterion as a reference benchmark.

Methods: In this prospective observational study, 128 adults aged 18-60 years with American Society of Anesthesiologists (ASA) physical status I-II who underwent elective surgery under general anaesthesia were evaluated. Recovery in all patients was assessed simultaneously using both the MAS and WFTS at predefined PACU intervals. The primary outcome was time to discharge readiness, defined as MAS ≥9 or WFTS ≥12 with no component score <1, against conventional 60-minute time-based discharge criteria. Secondary outcomes included comparison of recovery assessment between the two scoring systems and documentation of events associated with prolonged PACU stay.

Results: The mean age of participants was 33.83 ± 11.59 years, and 73 (57.0%) were female. Mean time to discharge readiness was 11.56 ± 5.05 minutes using MAS and 12.54 ± 6.55 minutes using WFTS, with no significant difference between the scores (t = 1.336, p = 0.183). No significant differences were observed in the proportions of patients meeting discharge criteria at any assessment interval (all p > 0.05). Both scoring systems identified discharge readiness significantly earlier than the conventional 60-minute criterion (p < 0.001).

Conclusions: MAS and WFTS demonstrated comparable performance in assessing PACU discharge readiness and identified discharge readiness significantly earlier than conventional time-based criteria, supporting a criteria-based approach to PACU discharge.

## Introduction

The post-anaesthesia care unit (PACU) plays a critical role in perioperative management by providing monitoring and support during recovery from anaesthesia. During this period, patients remain susceptible to physiological disturbances resulting from anaesthetic agents, surgical stress, and perioperative medications. Appropriate timing of PACU discharge is therefore essential to ensure patient safety while optimizing healthcare resource utilization [1-4]. Premature discharge may increase the risk of adverse events such as airway compromise, respiratory depression, hypoxaemia, haemodynamic instability, uncontrolled pain, and postoperative nausea and vomiting (PONV), whereas unnecessarily prolonged PACU stay may increase healthcare costs, delay patient flow, and reduce operating room efficiency [1,3,5].

Traditionally, PACU discharge decisions were based on predetermined observation periods. These time-based approaches evolved during periods when longer-acting anaesthetic techniques were more common and objective recovery assessment tools were less established [6-8]. Although simple to implement, fixed-duration observation protocols do not account for individual variability in recovery. Factors such as age, comorbidities, surgical procedures, and responses to anaesthetic drugs can significantly influence recovery trajectories, potentially leading to either premature discharge or unnecessary prolongation of PACU stay [3,5,8].

Advances in anaesthetic pharmacology and perioperative care have substantially improved postoperative recovery. The introduction of short-acting anaesthetic agents, enhanced monitoring technologies, objective neuromuscular recovery assessment, and multimodal analgesic strategies has enabled faster and more predictable emergence from anaesthesia [9-12]. These developments have highlighted the limitations of conventional time-based discharge criteria and increased the need for objective methods that assess actual recovery status rather than elapsed time. Consequently, standardized recovery scoring systems have become widely accepted tools for guiding PACU discharge decisions [1,3,4].

Among these tools, the Modified Aldrete Score (MAS) remains one of the most commonly used recovery assessment systems [6,13]. Owing to its simplicity, ease of application, and reproducibility, patients achieving a score of 9 or greater are generally considered suitable for PACU discharge [1,6,13]. However, MAS primarily evaluates physiological recovery and does not directly assess symptoms such as pain and PONV, which are important determinants of patient comfort and discharge readiness [14-17]. To address these limitations, White and colleagues developed a fast-track scoring system called the White Fast-Track Score (WFTS), originally introduced as Fast-Track Criteria (FTC) [17], which incorporates assessment of pain and emetic symptoms in addition to conventional physiological parameters, potentially providing a more comprehensive evaluation of postoperative recovery [17-19].

Several studies have demonstrated the utility of objective recovery scoring systems in facilitating safe and timely PACU discharge [8,20,21]. However, direct comparisons between the MAS and WFTS remain limited, particularly in the Indian healthcare setting, where patient profiles, healthcare resources, and PACU practices may differ from those reported in Western literature [20,22]. Furthermore, evidence comparing these scoring systems with conventional time-based discharge criteria remains limited.

Therefore, this study was undertaken to compare recovery assessment using the MAS and WFTS and to evaluate their performance against conventional time-based discharge criteria in adult patients undergoing elective surgery under general anaesthesia. We hypothesized that both scoring systems would provide an objective assessment of recovery and facilitate earlier identification of PACU discharge readiness compared with traditional fixed-duration observation protocols.

## Materials and methods

Study design and setting

This prospective observational study was conducted in the Department of Anaesthesiology and Intensive Care at the Acharya Shri Chander College of Medical Sciences and Hospital, Jammu, India. The study was approved by the Institutional Ethics Committee, Acharya Shri Chander College of Medical Sciences and Hospital (reference number: ASCOMS/IEC/2024/Meeting-II/15). The study was conducted over a period of 18 months, from June 2024 to November 2025. Written informed consent was obtained from all participants before enrollment.

Study objectives

The primary objective of the study was to compare the time to achieve PACU discharge readiness using the MAS [13] and WFTS [17] against conventional 60-minute time-based discharge criteria serving as the reference benchmark [8,20]. Secondary outcomes included comparison of recovery assessment between the two scoring systems and documentation of events associated with prolonged PACU stay.

Study population

Eligibility Criteria

All participants were assessed using both the MAS [13] and WFTS [17] during postoperative recovery. No separate study groups were created. Patients aged 18-60 years with American Society of Anesthesiologists (ASA) physical status I or II undergoing elective surgical procedures under general anaesthesia were included. Patients with ASA physical status III or IV, age below 18 years or above 60 years, significant systemic comorbidities such as chronic kidney disease, chronic liver disease, or coronary artery disease, neurological disorders affecting postoperative assessment including Alzheimer’s disease and cerebral palsy, inability to communicate adequately because of deafness, cognitive impairment, or mental disability, and those undergoing emergency surgical procedures were excluded. 

Sample Size Calculation

The sample size was calculated using G*Power software (version 3.1.9.7; Heinrich-Heine University, Düsseldorf, Germany) [23]. Based on findings from a previous study evaluating criteria-based PACU discharge [20], which demonstrated an approximate 10-minute reduction in PACU stay compared with conventional time-based discharge criteria, a minimum sample size of 108 participants was estimated at a significance level of 0.05 and a power of 80%. To compensate for potential dropouts and incomplete data, a total of 128 adult patients scheduled for elective surgery under general anaesthesia were included in the study.

**Figure 1:**
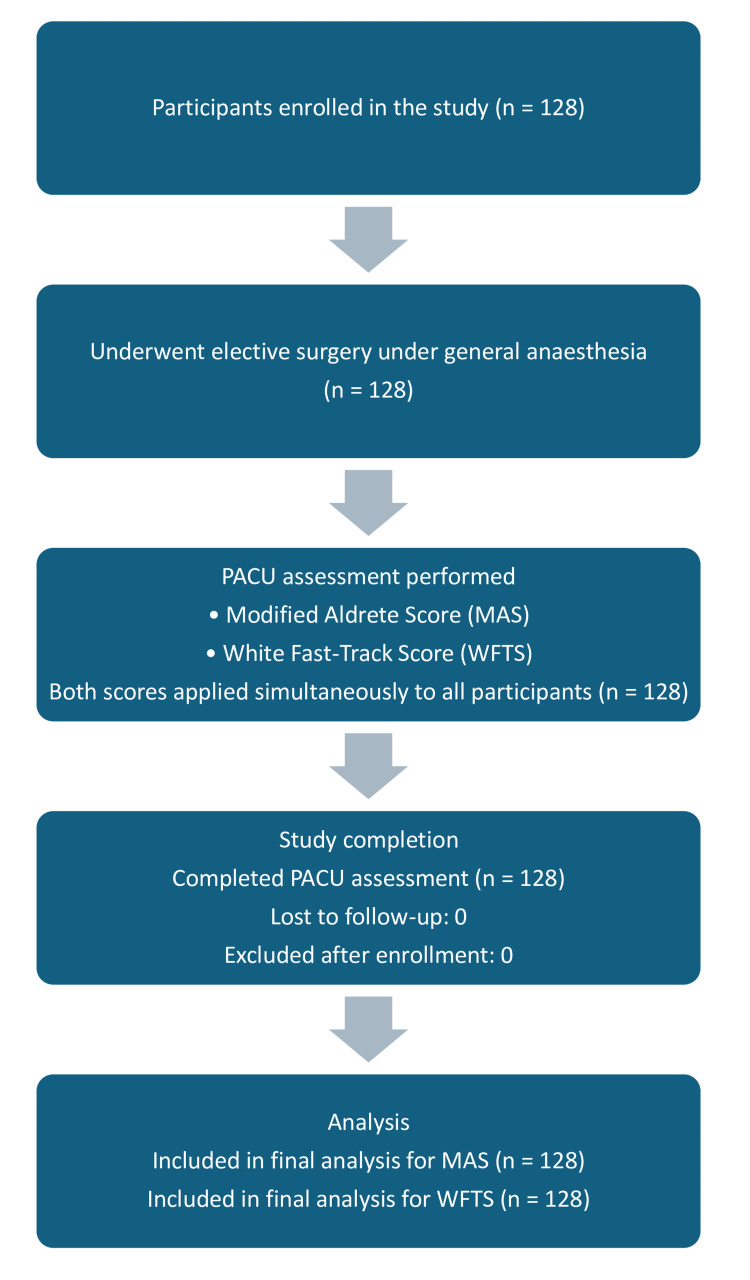
Flow diagram showing participant enrollment, completion of postoperative assessment, and inclusion in the final analysis. MAS: Modified Aldrete Score; PACU: post-anesthesia care unit; WFTS: White Fast-Track Score

The flow of participants through the study is illustrated in Figure 1.

Pre-anaesthetic evaluation and perioperative management

All patients underwent a comprehensive pre-anaesthetic evaluation one day before surgery, including detailed medical history, general physical examination, systemic examination, and airway assessment. Routine laboratory investigations and additional investigations, when indicated, were performed. Baseline demographic data, including age, sex, weight, height, and body mass index (BMI), were recorded. Patients were instructed to remain *nil per os* (nothing through the mouth) for at least eight hours before surgery and received oral alprazolam 0.25 mg and pantoprazole 40 mg on the night before surgery. On the day of surgery, an intravenous (IV) line was secured using an 18G (Gauge) or 20G cannula. Intravenous pantoprazole 40 mg and prophylactic antibiotics were administered according to institutional protocol.

Anaesthetic technique

Upon arrival in the operating room, standard monitoring was established, including electrocardiography (ECG), non-invasive blood pressure (NIBP), pulse oximetry (SpO₂), and heart rate monitoring. Physiological parameters were recorded at five-minute intervals throughout the procedure. All patients received general anaesthesia. Analgesia was provided with IV tramadol 1 mg/kg, and prophylaxis against postoperative nausea and vomiting was achieved using IV ondansetron 0.1 mg/kg. Tramadol was used as the institutional standard analgesic because stronger opioids such as fentanyl and morphine were not routinely available during the study period owing to regulatory licensing restrictions. No additional intraoperative opioids were administered. Anaesthesia was induced with propofol 2 mg/kg, followed by rocuronium 0.6-1.2 mg/kg to facilitate endotracheal intubation. Anaesthesia was maintained with a mixture of oxygen (33%), nitrous oxide (66%), and isoflurane (1%), supplemented with intermittent doses of rocuronium (0.1-0.2 mg/kg) as required. Intravenous paracetamol 15 mg/kg and diclofenac 1.5 mg/kg were administered for perioperative analgesia. Intravenous crystalloid fluids were administered according to the patient's body weight, fasting status, and intraoperative fluid requirements, with additional replacement provided as clinically indicated to maintain haemodynamic stability. End-tidal carbon dioxide was maintained between 35 and 45 mmHg. At the completion of surgery, neuromuscular recovery was assessed using quantitative Train-of-Four (TOF) monitoring. Residual neuromuscular blockade was reversed with IV neostigmine 0.05 mg/kg and glycopyrrolate 0.01 mg/kg. Tracheal extubation was performed after confirmation of adequate neuromuscular recovery (TOF ratio ≥0.9), together with satisfactory spontaneous ventilation and airway protective reflexes.

Perioperative data collection

The intraoperative variables recorded included duration of surgery (defined as the time from skin incision to final skin closure), duration of anaesthesia (defined as the time from induction to extubation), extubation time (defined as the time from discontinuation of anaesthetic agents to tracheal extubation), estimated blood loss, and any intraoperative anaesthetic or surgical complications.

PACU assessment

Following extubation, all patients were transferred to the PACU for postoperative monitoring. Continuous monitoring using ECG, pulse oximetry, and non-invasive blood pressure measurement was maintained throughout the recovery period. Both the MAS [13] and WFTS [17] were recorded five minutes after PACU admission. Subsequently, scores were assessed every five minutes during the first 20 minutes and every 10 minutes thereafter until discharge criteria were achieved. The MAS and WFTS were assessed simultaneously at each time point by the same anaesthesiologist, who was trained in the application of both scoring systems. Blinding of the assessor was not feasible because both scoring systems were derived from the same contemporaneous clinical assessment of each patient. To minimize measurement bias, predefined scoring criteria from the original published descriptions were applied consistently throughout the study. Discharge readiness according to the MAS was defined as a score of nine or greater. Discharge readiness according to WFTS was defined as a total score of 12 or greater, with no individual parameter score less than one.

The MAS was originally described by Aldrete as a clinical recovery scoring system for assessing readiness for PACU discharge [13], while the WFTS was developed by White et al. for rapid evaluation of recovery after anaesthesia [17]. Conventional time-based discharge readiness was defined as completion of a fixed 60-minute observation period in the PACU according to institutional practice [8,20]. Time to discharge readiness was calculated from PACU admission until the first assessment at which discharge criteria were fulfilled. Any postoperative adverse event, including PONV, significant pain requiring rescue analgesia, respiratory events, haemodynamic instability, or any other event resulting in prolonged PACU stay, was documented. Pain assessment, which forms part of the WFTS, was performed using the Numeric Rating Scale (NRS). Pain severity was categorized as mild (scores 1-3), moderate (scores 4-6), or severe (scores 7-10) [15,24]. Direct comparison of absolute MAS and WFTS scores was performed descriptively, as the two scoring systems use different scoring structures and components. Discharge readiness was assessed according to the predefined threshold criteria for each scoring system.

Statistical analysis

Data were analysed using IBM SPSS Statistics for Windows, version 20 (Released 2011; IBM Corp., Armonk, New York, United States). Continuous variables were expressed as mean ± standard deviation (SD) or median (interquartile range (IQR)), as appropriate, whereas categorical variables were presented as frequencies and percentages. The normality of continuous variables was assessed using the Kolmogorov-Smirnov test. Normally distributed paired continuous variables were analysed using the paired Student's t-test, whereas the one-sample t-test was used where appropriate. Categorical variables were analysed using the Z-proportion test. All statistical tests were two-tailed, and a p-value <0.05 was considered statistically significant.

## Results

Baseline characteristics

A total of 128 patients were included in the study. The mean age of the study population was 33.83 ± 11.59 years (range: 18-60 years). Most patients belonged to the age group of 31-40 years (n=36, 28.1%), followed by the age group of 21- 30 years (n=34, 26.6%). The majority of the study population was female (n=73, 57.0%), whereas 55 (43.0%) were male. The majority of patients were classified as ASA grade I (n=82, 64.1%), while 46 (35.9%) were ASA grade II. Most patients (n=82, 64.1%) had no associated comorbidities. Among those with comorbid conditions, hypertension was the most common (n=22, 17.2%), followed by hypothyroidism (n=12, 9.3%) and diabetes mellitus (n=11, 8.6%). Hyperthyroidism was present in only one (0.8%) patient (Table 1). The mean height, weight, and BMI of the study population were 165.34 ± 5.87 cm, 65.98 ± 10.50 kg, and 24.12 ± 3.19 kg/m², respectively.

**Table 1:** Comorbidity pattern in the study population (N=128)

Co-morbidity	Frequency (Percentage)
Diabetes Mellitus	11 (8.6%)
Hypertension	22 (17.2%)
Hyperthyroidism	1 (0.8%)
Hypothyroidism	12 (9.3%)
None	82 (64.1%)

Perioperative characteristics

**Figure 2:**
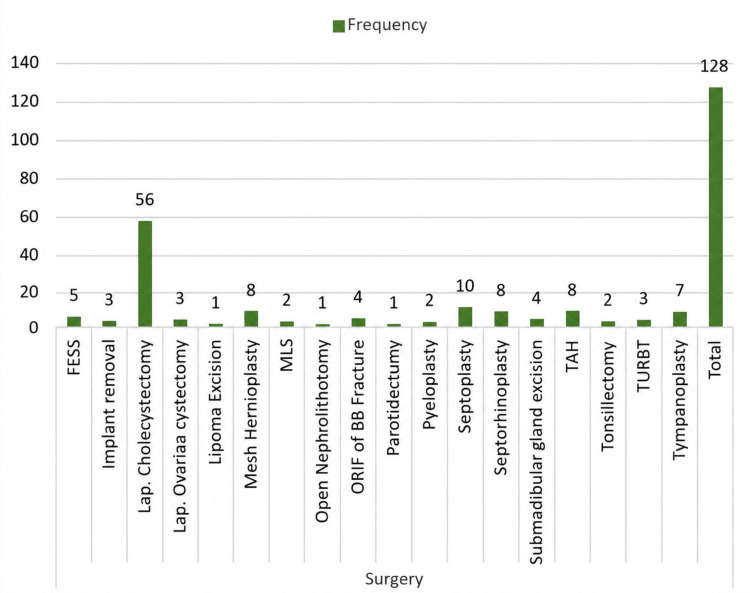
Types of surgeries performed in study population (N=128) BB: both bone; FESS: functional endoscopic sinus surgery; Lap.: laparoscopic; MLS: microlaryngeal surgery; ORIF: open reduction and internal fixation; TAH: total abdominal hysterectomy; TURBT: transurethral resection of bladder tumor

Baseline haemodynamic parameters were within normal physiological limits. The mean systolic blood pressure was 123.56 ± 9.63 mmHg, mean diastolic blood pressure was 72.14 ± 9.40 mmHg, mean heart rate was 78.94 ± 9.94 beats/min, and mean oxygen saturation (SpO₂) was 99.05 ± 0.79%. The mean duration of surgery was 53.57 ± 27.44 minutes, while the mean duration of anaesthesia was 72.54 ± 29.65 minutes. The mean extubation time was 6.45 ± 1.34 minutes. A variety of surgical procedures were included in the study. Laparoscopic cholecystectomy was the most common procedure, accounting for 56 (43.8%) of all surgeries. Other frequently performed procedures included septoplasty (n=10, 7.8%), mesh hernioplasty (n=8, 6.3%), total abdominal hysterectomy (n=8, 6.3%), septorhinoplasty (n=8, 6.3%), and tympanoplasty (n=7, 5.5%) (Figure 2).

Postoperative recovery events

Postoperative recovery was uneventful in the majority of patients. Overall, 24 patients (18.8%) required rescue postoperative analgesia, whereas 104 (81.2%) achieved satisfactory pain control without additional analgesic intervention. Postoperative antiemetic therapy was required in 31 patients (24.2%), while 97 (75.8%) did not require antiemetic intervention. No patient experienced major postoperative adverse events, including airway compromise, oxygen desaturation requiring intervention, reintubation, or unplanned intensive care admission. Furthermore, no adverse event resulted in prolonged PACU stay or delayed discharge beyond the institutional observation period.

Comparison of discharge readiness using MAS and WFTS

At five minutes, 33 (25.8%) patients achieved MAS discharge criteria compared with 32 (25.0%) achieving WFTS criteria. At 10 minutes, discharge readiness was observed in 37 (28.9%) and 35 (27.3%) patients according to MAS and WFTS, respectively. By 15 minutes, 45 (35.2%) patients met MAS criteria compared with 39 (30.5%) meeting WFTS criteria. The remaining patients achieved discharge readiness at subsequent assessment intervals. There was no statistically significant difference between the proportions of patients achieving MAS and WFTS at any time interval (all Z-proportion values and p-values are presented in Table 2). All participants achieved discharge criteria according to both scoring systems within the observation period.

**Table 2:** Proportion of patients meeting MAS and WFTS discharge scores across time intervals (N=128) MAS: Modified Aldrete Score; WFTS: White Fast-Track Score

Time	MAS achieved, n (%)	WFTS achieved, n (%)	Z-proportion	p-value
5 minutes	33 (25.8%)	32 (25.0%)	0.140	0.886
10 minutes	37 (28.9%)	35 (27.3%)	0.280	0.781
15 minutes	45 (35.2%)	39 (30.5%)	0.800	0.424
20 minutes	12 (9.4%)	16 (12.5%)	0.800	0.423
30 minutes	1 (0.8%)	5 (3.9%)	1.660	0.097
40 minutes	0 (0.0%)	1 (0.8%)	1.000	1.000

Time to achieve discharge readiness

The mean time required to achieve discharge criteria was 11.56 ± 5.05 minutes using the MAS and 12.54 ± 6.55 minutes using WFTS. Although the MAS identified discharge readiness approximately one minute earlier than WFTS, the difference was not statistically significant (t = 1.336, p = 0.183).

Recovery scores during PACU stay

Both the MAS and WFTS demonstrated progressive improvement throughout the PACU stay. At five minutes, the mean MAS and WFTS scores were 8.04 ± 1.29 and 11.13 ± 1.51, respectively. Corresponding mean scores at 10, 15, 20, and 30 minutes were 8.41 ± 1.25 and 11.52 ± 1.47, 9.28 ± 0.97 and 12.34 ± 1.15, 9.46 ± 0.66 and 12.64 ± 1.09, and 9.67 ± 0.52 and 12.81 ± 0.91, respectively. Because MAS and WFTS use different scoring ranges and component structures, comparisons of absolute score values were descriptive only (Table 3).

**Table 3:** Comparison of MAS and WFTS scores at different time intervals MAS: Modified Aldrete Score; WFTS: White Fast-Track Score

Time	MAS, mean ± SD	WFTS, mean ± SD	Paired t-value	p-value
5 minutes	8.04 ± 1.29	11.13 ± 1.51	17.613	<0.001
10 minutes	8.41 ± 1.25	11.52 ± 1.47	15.727	<0.001
15 minutes	9.28 ± 0.97	12.34 ± 1.15	15.671	<0.001
20 minutes	9.46 ± 0.66	12.64 ± 1.09	9.468	<0.001
30 minutes	10.00 ± 0.00	12.83 ± 0.98	2.668	0.044
40 minutes	-	13.00 ± 0.00	-	-

Comparison with conventional time-based discharge criteria

When compared with the conventional fixed 60-minute PACU observation protocol [8,20], both scoring systems identified discharge readiness substantially earlier. The mean time to achieve discharge readiness was 11.56 ± 5.05 minutes using the MAS and 12.54 ± 6.55 minutes using WFTS. To quantify the difference between criteria-based discharge readiness and the conventional fixed 60-minute PACU observation protocol, a one-sample t-test was performed using 60 minutes as the reference value. Both MAS and WFTS demonstrated significantly shorter times to discharge readiness than the conventional protocol (Table 4). No patient experienced an event resulting in prolonged PACU stay, and no participant required extended observation beyond the institutional discharge protocol.

**Table 4:** Comparison of time to achieve discharge readiness using MAS, WFTS, and conventional time-based discharge criteria.

Score	Number of patients	Time to achieve discharge readiness, mean ± SD	t-value	p-value
Modified Aldrete Score (MAS)	128	11.563 ± 5.049 minutes	108.538	<0.001
White Fast-Track Score (WFTS)	128	12.539 ± 6.551 minutes	81.970	<0.001
Conventional Time-Based Discharge Criteria	128	60 minutes	-	-

## Discussion

The present prospective observational study compared recovery assessment using the MAS and WFTS with conventional time-based discharge criteria in adult patients undergoing elective surgery under general anaesthesia. The principal finding of this study was that both scoring systems identified discharge readiness substantially earlier than the conventional 60-minute PACU observation period. Furthermore, no statistically significant difference was observed between MAS and WFTS regarding the proportion of patients achieving discharge readiness or the mean time required to attain discharge criteria.

Appropriate discharge from the PACU is essential to ensure patient safety while optimizing perioperative resource utilization. Traditionally, discharge decisions were based largely on predetermined observation periods. However, advances in anaesthetic techniques, use of short-acting agents, multimodal analgesia, and improved monitoring have resulted in more predictable recovery patterns, questioning the continued relevance of fixed-duration discharge protocols. The findings of the present study support the increasing use of objective recovery assessment tools to provide individualized and efficient discharge decisions.

The demographic characteristics of the study population were comparable with previous studies evaluating PACU recovery and discharge criteria. The mean age of participants was 33.83 ± 11.59 years, with most patients belonging to the young and middle-aged adult population. Similar age distributions have been reported by Truong et al. [8] and Jain et al. [20], although slightly higher mean ages were observed in their cohorts, likely due to differences in patient selection and surgical case mix. Female patients constituted 73 (57.0%) of the study population, which was comparable to findings reported by Taesiri et al. [21]. These similarities indicate that the study population represented patients commonly undergoing elective surgical procedures under general anaesthesia.

Most participants were ASA physical status I or II and had limited comorbidities. Hypertension, hypothyroidism, and diabetes mellitus were the most frequently encountered medical conditions. The predominance of low-risk patients is consistent with previous studies assessing structured PACU discharge criteria and supports the applicability of these findings in routine elective surgical practice. However, extrapolation to higher-risk patients should be performed cautiously, as patients with severe systemic disease were excluded.

The perioperative characteristics observed in the present study were also comparable with previously reported literature. The mean duration of surgery was 53.57 ± 27.44 minutes, while the mean duration of anaesthesia was 72.54 ± 29.65 minutes. Extubation occurred at a mean time of 6.45 ± 1.34 minutes following discontinuation of anaesthetic agents. Similar rapid emergence and recovery patterns have been described by Song et al., reflecting the benefits of contemporary anaesthetic practices [12]. These findings further highlight the limitations of relying solely on fixed-duration recovery protocols [9-11].

Postoperative pain and nausea remain important contributors to delayed recovery and prolonged PACU stay. In the present study, 24 (18.8%) patients required postoperative opioid analgesia, while 31 (24.2%) patients required antiemetic therapy. Although the overall incidence was relatively low, these factors influenced recovery assessment, particularly with WFTS, which incorporates pain and postoperative emetic symptoms into discharge evaluation. These findings emphasize the importance of including patient-centred recovery parameters along with physiological stability when determining discharge readiness. Previous studies have similarly identified pain and postoperative nausea and vomiting as important determinants of recovery quality [14-16,24].

A key finding of this study was the absence of a statistically significant difference between MAS and WFTS in determining discharge readiness. The mean time to achieve discharge criteria was 11.56 ± 5.05 minutes using MAS and 12.54 ± 6.55 minutes using WFTS (t = 1.336, p = 0.183). Although MAS identified discharge readiness approximately one minute earlier, this difference was not clinically or statistically significant. Similarly, no significant difference was observed in the proportion of patients meeting discharge criteria at different assessment intervals. These findings suggest that both scoring systems are effective tools for assessing recovery readiness in low-risk patients undergoing elective surgery under general anaesthesia.

The present findings are broadly consistent with previous studies evaluating structured recovery scoring systems, which have reported comparable performance of MAS and WFTS in assessing discharge readiness [17,19,20,25]. WFTS may offer a broader assessment of recovery by incorporating pain and PONV; however, these factors did not result in a clinically meaningful delay in discharge readiness in the present study. This may be explained by the relatively low incidence of significant postoperative symptoms, likely related to standardized multimodal analgesic and antiemetic strategies.

Importantly, both scoring systems identified discharge readiness considerably earlier than the conventional time-based discharge protocol. Patients achieved discharge criteria at approximately 12 minutes using objective assessment tools compared with the fixed 60-minute observation period. These findings indicate that many patients may remain in the PACU longer than necessary when discharge decisions are based only on elapsed time rather than individual recovery status.

From a clinical perspective, implementation of objective discharge scoring systems may improve PACU workflow by facilitating earlier identification of appropriately recovered patients, reducing unnecessary occupancy, and improving utilization of recovery resources. These benefits are particularly relevant in healthcare settings where PACU capacity and staffing resources are limited. Structured discharge criteria may therefore enhance perioperative efficiency while maintaining objective assessment standards [11,26-28].

Although both scoring systems demonstrated comparable performance, each has specific advantages. The MAS is simple, widely applicable, and primarily evaluates physiological recovery parameters. In contrast, WFTS provides a broader assessment by incorporating pain and PONV, which are important components of patient comfort and recovery quality. Therefore, WFTS may be particularly useful in ambulatory surgery and enhanced recovery pathways where symptom control contributes significantly to discharge decisions.

Strengths, limitations, and future recommendations

The strengths of the present study include its prospective design, standardized anaesthetic management, inclusion of patients undergoing a variety of elective surgical procedures, and simultaneous application of both recovery scoring systems in all patients. Additionally, recovery assessments were performed using a standardized protocol, reducing variability in data collection.

Several limitations should be acknowledged. First, this was a single-centre observational study with a relatively modest sample size, which may limit the generalizability of the findings and the ability to detect smaller differences between recovery assessment methods. The observational design also limits the ability to establish causal relationships, and residual confounding factors cannot be completely excluded. Second, only ASA physical status I and II patients undergoing elective surgical procedures were included; therefore, the applicability of these findings to higher-risk patients, emergency surgeries, and major procedures remains uncertain. Furthermore, the inclusion of different types of elective surgical procedures may have introduced variability in postoperative recovery profiles, as differences in surgical complexity and duration may influence PACU recovery times.

Third, recovery assessments were performed by a single trained anaesthesiologist, and blinding was not feasible because both scoring systems were derived from the same contemporaneous clinical assessment. Although standardized scoring criteria were applied consistently throughout the study, observer bias cannot be completely excluded. Fourth, the anaesthetic protocol used tramadol instead of more potent intraoperative opioids such as fentanyl or morphine because of institutional regulatory licensing constraints. As recovery characteristics may differ in other opioid regimens, caution should be exercised when generalizing these findings to institutions where fentanyl- or morphine-based anaesthesia is routinely used.

Fifth, nitrous oxide was routinely used for maintenance of anaesthesia. Although nitrous oxide has been associated with an increased incidence of postoperative nausea and vomiting, all participants received the same standardized anaesthetic regimen together with prophylactic ondansetron. Nevertheless, the influence of anaesthetic practices involving nitrous oxide on recovery profiles cannot be completely excluded, and the findings should be interpreted with caution in settings where nitrous oxide is not routinely used. Sixth, Bispectral Index (BIS) monitoring was not used to standardize the depth of anaesthesia. However, all patients received a standardized anaesthetic protocol, and quantitative TOF monitoring was used to confirm adequate neuromuscular recovery before extubation, thereby reducing the likelihood of systematic bias related to residual neuromuscular blockade.

Importantly, this study evaluated early identification of PACU discharge readiness using the MAS and WFTS in a low-risk elective surgical population and did not evaluate actual early PACU discharge or establish the safety of accelerated discharge pathways. Therefore, these findings should be interpreted as demonstrating earlier recognition of discharge readiness rather than definitive evidence supporting safe reduction in PACU discharge times. The conventional 60-minute time-based discharge criterion was used only as an institutional reference benchmark and not as a separate intervention or comparison group. Finally, post-PACU safety outcomes, including patient satisfaction, unplanned PACU readmission, escalation of care, delayed postoperative complications, and a formal cost-effectiveness analysis of criteria-based discharge, were not evaluated and should be addressed in future studies.

Future multicentre studies involving larger and more diverse patient populations are required to validate these findings. Inclusion of elderly patients, higher ASA grades, emergency procedures, and enhanced recovery pathways would provide further insight into the performance of recovery scoring systems across broader clinical settings. Studies evaluating economic outcomes and patient-reported recovery measures may also help determine the overall impact of criteria-based PACU discharge strategies.

## Conclusions

Both the MAS and WFTS demonstrated comparable performance in assessing postoperative recovery and identified PACU discharge readiness earlier than the conventional 60-minute time-based criterion. No significant difference was observed between the two scoring systems, indicating comparable performance in evaluating recovery following general anaesthesia. While MAS provides a simple assessment of physiological recovery, WFTS offers a broader evaluation by incorporating pain and postoperative emetic symptoms. The use of objective, criteria-based recovery assessment systems may support individualized evaluation of discharge readiness and provide a structured approach to PACU recovery assessment. These findings support the role of structured recovery scoring systems for assessing PACU discharge readiness in appropriately selected low-risk elective surgical patients.
